# Additional role of ECC in the detection and treatment of cervical HSIL

**DOI:** 10.3389/fmed.2023.1206856

**Published:** 2023-09-13

**Authors:** Li Sijing, Jia Ying, Wu Jing, Li Xiaoge, Luo Ming, Duan Zhaoning

**Affiliations:** Department of Gynecology, The First Affiliated Hospital of Chongqing Medical University, Chongqing, China

**Keywords:** high-grade squamous intraepithelial lesion, endocervical curettage, colposcopic targeted biopsy, loop electrosurgical excision procedure, cervical lesions

## Abstract

**Objective:**

To probe into the additional role of ECC in the detection of cervical HSIL. The primary objective was to risk-stratify HSIL patients according to ECC so as to provide clinical suggestions for subsequent treatment.

**Methods:**

Retrospective analysis of medical records for patients with HSIL. All patients underwent both ECC and cervical biopsy. According to the results of colposcopic targeted biopsy and ECC, the patients were divided into three groups: (1) ECC negative group (those whose colposcopic targeted biopsy indicated HSIL, but ECC indicated LSIL or chronic inflammation); (2) Only the ECC positive group (those whose ECC suggested HSIL, but colposcopic targeted biopsy showed LSIL or chronic inflammation); (3) ECC and biopsy positive group (those whose ECC and targeted biopsy were both HSIL). Chi-square test was used to analyze the differences of lesion residue and biopsy results after LEEP amongst the three groups.

**Results:**

A total of 1,146 medical records were analyzed. The diagnostic accuracy of ECC combined with colposcopic targeted biopsy for HSIL was higher than that of colposcopic biopsy alone (72.43% vs. 67.54%). When ECC indicated HSIL, the coincidence rate of ECC combined with colposcopic targeted biopsy and the histological pathology of LEEP was 86.25%, and the proportion of residual lesions after LEEP was 41.43%. When ECC and targeted biopsy both indicated HSIL, HSIL or worse lesions were confirmed in 90.68% of patients after surgery. Of these, 10.77% were confirmed as cervical invasive carcinoma. Moreover, the positive rate of LEEP resection margin and postoperative ECC in these patients was 43.48%.

**Conclusion:**

ECC can improve the detection rate of cervical HSIL and reduce missed diagnosis. Also ECC can help clinicians predict the proportion of residual lesions after LEEP. This provides the gynecologists with a reference for the need to increase the depth of the procedure and the need to perform ECC for the residual cervical canal.

## 1. Introduction

Cervical cancer is one of the most common gynecological malignancies, and its mortality rate ranks fourth among female malignancies worldwide (1). High-grade squamous intraepithelial lesion (HSIL) is a precancerous lesion closely related to cervical cancer. In November 2020, the World Health Organization released the global strategy to accelerate the elimination of cervical cancer, calling for the goal of reducing the incidence of cervical cancer to less than 4 per 100,000 by the end of the century (2). As the only disease with a complete cancer prevention and treatment strategy, the diagnosis and treatment of cervical precancerous lesions is a key step toward achieving the goal of “eliminating cervical cancer” (3). Cervical biopsy is the “gold standard” for the diagnosis of precancerous lesions of cervical cancer. However, the diagnostic accuracy of colposcopic directed biopsy varies with age, menopausal status, and the type of cervical transformation zone Ren et al. (4). When patients undergo colposcopy, if the lesion in the cervical canal cannot be excluded, endocervical curettage (ECC) should be performed at the same time (5). ECC can compensate for the defects of point biopsy and improve the sensitivity of colposcopy. However, due to limited sampling, ECC has a certain rate of unqualified specimens and the consistency amongst different observers is poor (6, 7). Therefore, the clinical application of ECC is still controversial.

Here we retrospectively analyzed the medical records of patients with HSIL who underwent loop electrosurgical excision procedure (LEEP). To probe into the additional role of ECC in the detection of cervical HSIL. The primary objective was to risk-stratify HSIL patients according to ECC so as to provide clinical suggestions for subsequent treatment. The result showed great potential for the protection of patient fertility.

## 2. Materials and methods

### 2.1. Study population

Complete medical records for patients who underwent a loop electrosurgical excision procedure for ECC combined with colposcopic targeted biopsy indicating cervical HSIL in the Cervical Clinic of the First Affiliated Hospital of Chongqing Medical University from January 1, 2020, to December 31, 2021, were retrospectively analyzed. Data included the patient’s basic information (age, address, education level, age of first sexual intercourse, gestational number), thinprep cytology test (TCT), HPV infection, and clinical symptoms (such as abnormal vaginal bleeding, abnormal vaginal discharge, vulvar pruritus, etc).

The inclusion criteria were as follows: (1) cervical biopsy and ECC were performed at the same time under colposcopy; (2) ECC combined with cervical biopsy indicating cervical HSIL; and (3) patients who underwent LEEP operation at the First Affiliated Hospital of Chongqing Medical University. The exclusion criteria were as follows: (1) the patient’s ECC samples were unsuitable, for example if the samples obtained from ECC were only mucus, blood clots, inflammatory cells or endometrium, with no cervical canal cells or tissue; (2) The patient had incomplete medical records.

This study was approved by the hospital ethics review committee. The study was performed according to the Declaration of Helsinki (as revised in 2013) after approval was granted by the Ethics Committee of the first affiliated hospital of Chongqing medical university (K2023-069). The study was conducted without informed consent because it was a retrospective study.

### 2.2. ECC combined with colposcopic targeted biopsy

Patients were evaluated colposcopically by professional colposcopists with more than 5 years of working experience. The patient’s cervix was fully exposed during the examination, and the condition of the cervix was assessed after the surface of the cervix was wiped with saline. The cervical lesions were initially evaluated by acetic acid staining and iodine staining, and targeted biopsy were performed where cervical lesions were suspected. When colposcopy was performed, if there was a suspected lesion in the cervical canal, ECC was performed to confirm the situation in the cervical canal. All 1,146 patients included in this study underwent ECC combined with biopsy.

For ECC, a sharp Kevorkian curette without basket was placed inside the endocervical canal. Gentle pressure was applied at its tip, and the curette was moved back and forth along the length of the endocervix while being rotated in a circular fashion to sample the entire circumference of the canal. The extension of sampling to lesions external to the cervical os was avoided during the procedure to minimize contamination with ectocervical tissue. A rapid spinning motion was exerted when removing the curette from the endocervical canal, trapping all tissue and cellular materials in the curette chamber. To retrieve any remaining curettage material, Singly or Campion forceps could be introduced into the canal. In women with severe cervical stenosis, a cytobrush (Cervix-Brush) or Wallach broom was used to provide specimens to evaluate the endocervical canal.

### 2.3. Surgical procedures

LEEP was performed according to the recommended procedure (8). Local anesthesia was administered before surgery. After satisfactory anesthesia, the cervix was dilated, and the lesion was labeled with Lugol’s iodine solution. A LEEP knife was used for annular resection of the lesion, and the length of resection was determined according to the transformation area. The external incisal margin of the cervix was usually 3–5 mm beside the lesion. In the case of a cervical type 1 transformation area, the depth of coning was 1 cm. Type II and III transformation areas were excised with margins of at least 1.5 cm and 2 cm, respectively. Immediately after the cervical coning was completed, the remaining cervical canal was scraped with a curette. Electrocoagulation and hemostasis were utilized during the operation. Conical specimens were marked at 12 points after surgery, and the ECC tissue and conical tissue were sent for pathological examination.

### 2.4. Interpretation of the pathological findings

The pathological diagnoses were made by a pathologist after examining the tissue sections, and the senior physician re-examined the tissue sections to confirm the diagnosis. Some difficult diseases were further confirmed by immunohistochemical staining. The pathological findings of normal and chronic inflammation were classified as “no abnormality,” and lesions of HSIL and above were classified as HSIL+. The final result was the most severe results from both targeted biopsy and ECC. The results of the LEEP postoperative examination were used as the “gold standard” to determine whether the targeted biopsy and ECC were consistent with the postoperative results. The ratio of additional HSIL detected by ECC was defined as the additional detection rate of ECC, i.e., the ECC diagnosis was HSIL, while the targeted biopsy diagnosis was low-grade squamous intraepithelial lesion (LSIL) or normal, divided by the total number of patients enrolled.

### 2.5. Grouping standard

Patients were grouped according to the pathological results of ECC and targeted biopsy, the enrolled patients were divided into three groups: (1) ECC negative group: those whose colposcopic targeted biopsy indicated HSIL, but ECC indicated LSIL or chronic inflammation. It is expressed as “ECC(−)&Biopsy(+)” in the following text. (2) Only the ECC positive group: those whose ECC suggested HSIL, but colposcopic targeted biopsy showed LSIL or chronic inflammation. It is expressed as “ECC(+)&Biopsy(−)” in the following text. (3) ECC and biopsy positive group: those whose ECC and targeted biopsy were both HSIL. It is expressed as “ECC(+)&Biopsy(+)” in the following text.

### 2.6. Statistical method

SPSS 23.0 software was used for data analysis. Data with a normal distribution [mean ± standard deviation (*X* ± *s*)] were considered to not conform to the normal distribution of measurement data using a nonparametric test. Count data were expressed as rate (%). The chi-square test was used for comparisons between the groups and the rank sum test was used for ranked data. *p* < 0.05 was considered statistically significant.

## 3. Results

### 3.1. Patient characteristics

**Figure 1:**
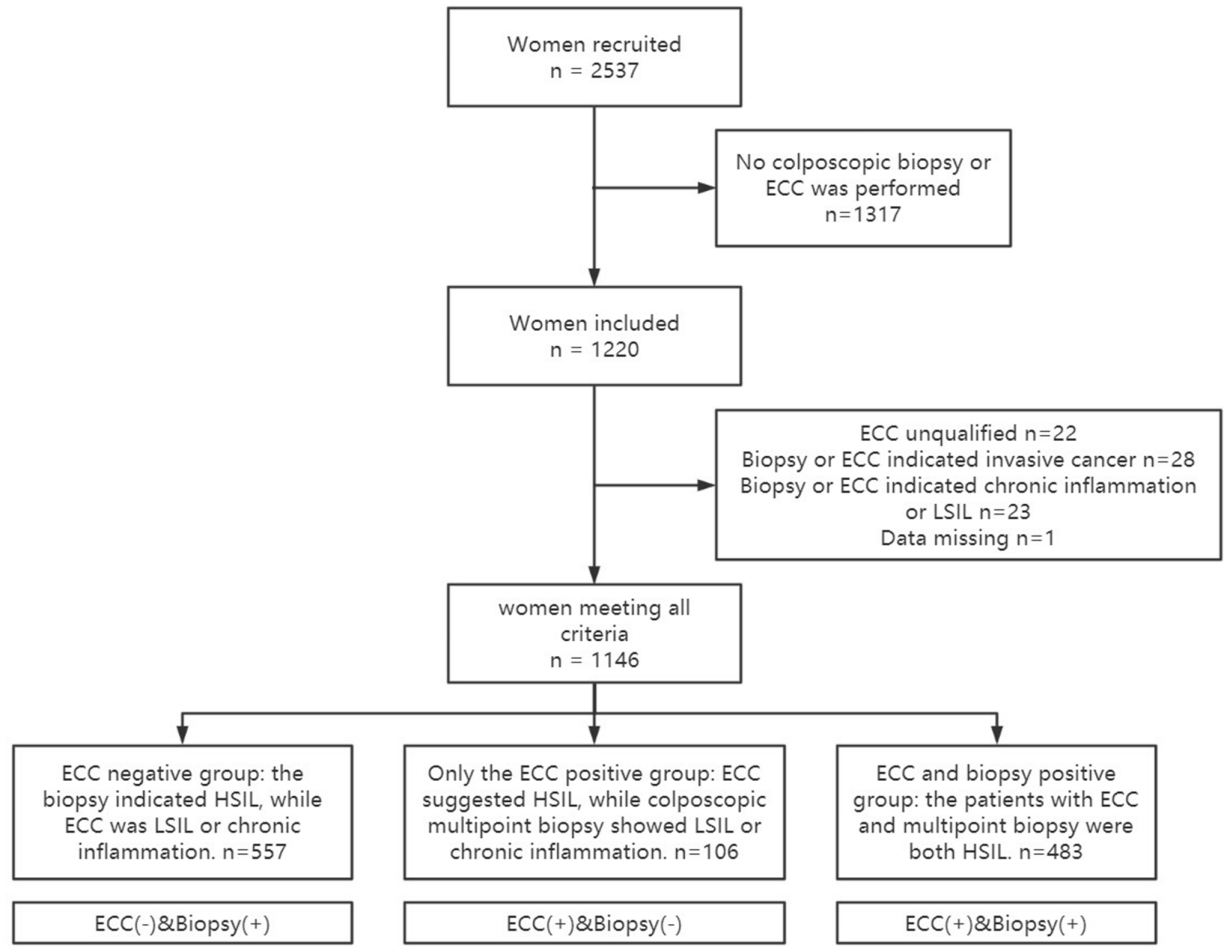
The inclusion and exclusion criteria.

A total of 1,146 patients were included in this study, and the specific inclusion and exclusion criteria are shown in Figure 1. Of these, 557 patients (48.60%) were ECC negative, 106 patients (9.25%) were only ECC suggestive of cervical HSIL, and 483 patients (42.15%) were both biopsy and ECC suggestive of HSIL. The baseline data of the patients in the three groups are shown in Table 1.

**Table 1:** The baseline data of the patients in the three groups.

	ECC(−)&Biopsy(+)	ECC(+)&Biopsy(−)	ECC(+)&Biopsy(+)	*X*^2^	*p*
Median age (inter-quartile range)	41 (34–49)	47.5 (42–54)*	46 (38–52)*	47.672	<0.001
*Age of first intercourse*					
≤18	117 (22.76)	21 (21.43)	95 (20.65)	0.642	0.726
>18	397 (77.24)	77 (78.57)	365 (79.35)		
*Gravidity*					
<3	208 (37.61)	28 (26.67)	151 (31.39)	7.192	0.027
≥3	345 (62.39)	77 (73.33)	330 (68.61)		
*Parity*					
<3	506 (91.50)	99 (94.29)	440 (91.29)	1.054	0.59
≥3	47 (8.50)	6 (5.71)	42 (8.71)		
*HPV infection*					
None	16 (2.95)	2 (1.94)	11 (2.37)	0.532	0.766
HPV16 co-infection	176 (32.41)	42 (40.78)	212 (45.59)*	18.544	<0.001
HPV18 co-infection	47 (8.66)	3 (2.91)	28 (6.02)	5.597	0.061
Other Hr-HPV infection	304 (55.99)	56 (54.37)	214 (46.02)*	10.291	0.006
*TCT*					
<ASC-H	314 (75.85)	58 (69.05)	179 (48.51)*★	64.153	<0.001
≥ASC-H	100 (24.15)	26 (30.95)	190 (51.49)*★		
*Transformation zone (TZ)*					
TZ 1	81 (15.28)	1 (1.00)*	24 (5.39)*	46.384	<0.001
TZ 2	76 (14.34)	8 (8.00)*	45 (10.11)*		
TZ 3	373 (70.38)	91 (91.00)*	376 (84.49)*		
*Colposcopy impression*					
Normal	12 (2.15)	3 (2.83)*	7 (1.45)*★	48.744	<0.001
LSIL	165 (29.62)	44 (41.51)*	73 (15.11)*★		
HSIL	380 (68.22)	59 (55.66)*	403 (83.44)*★		
*Clinical symptoms*					
None	398 (71.45)	81 (76.42)	344 (71.22)	1.228	0.541
Abnormal vaginal bleeding	100 (17.95)	16 (15.09)	97 (20.08)	1.717	0.424
Abnormal vaginal discharge	86 (15.44)	11 (10.38)	66 (13.66)	2.084	0.353

*Compared with ECC negative group, the difference was statistically significant (*p* < 0.05).

★Compared with only the ECC positive group, the difference was statistically significant (*p* < 0.05).

Age, gravidity, cervical cytological results, HPV infection, type of transformation zone, and colposcopy impression were statistically significantly different between the three groups (Table 1). After grouping comparison, it was found that patients in “ECC(−)&Biopsy(+)” group were significantly younger than those in the ECC positive groups (*p* < 0.001). There was no difference in age between the “ECC(+)&Biopsy(−)” and “ECC(+)&Biopsy(+)” (*p* = 0.201). The proportion of patients with HPV 16 infection was lower in “ECC(−)&Biopsy(+)” compared with “ECC(+)&Biopsy(+)” (*p* < 0.001). In the “ECC(+)&Biopsy(+),” the proportion of HSIL diagnosed by colposcopy and TCT ≥ ASC-H was higher than that in the other two groups (*p* < 0.05). There were fewer patients with type 3 transformation zone in “ECC(−)&Biopsy(+)” than the ECC positive groups (*p* < 0.001).

### 3.2. Diagnostic accuracy of colposcopic targeted biopsy and ECC combined with targeted biopsy for cervical HSIL

The coincidence rate of the colposcopic targeted biopsy and the cervical conization specimens was 67.54%. The consistency rate of ECC combined with colposcopic targeted biopsy and histological pathology of surgery was 72.43% (Table 2). The difference in the diagnostic accuracy of cervical HSIL between the two examination methods was statistically significant (*p* = 0.011, *p* < 0.05). In 106 patients biopsy did not indicate HSIL but ECC indicated HSIL (Table 3). These patients underwent LEEP procedure. Postoperatively 70 patients were found to be HSIL+ (HSIL and higher grade lesions). ECC prevented these 70 HSIL+ patients from being missed; the extra detection rate of cervical HSIL+ by ECC was 6.11% (70/1146).

**Table 2:** The consistency rate of ECC combined with colposcopic targeted biopsy and histological pathology of LEEP.

Method		Histological pathology of LEEP			The coincidence rate(%)	*X*^2^	*p*
		Chronic inflammation	LSIL	HSIL+			
Targeted biopsy	Chronic inflammation	7	9	36	67.54	6.513	0.011
	LSIL	13	7	34			
	HSIL	130	150	760			
Targeted biopsy + ECC	Chronic inflammation	0	0	0	72.43		
	LSIL	0	0	0			
	HSIL	150	166	830			

The difference in the diagnostic accuracy of cervical HSIL between the two examination methods was statistically significant (*p* = 0.011, *p* < 0.05).

**Table 3:** Comparison of the consistency rate among the three groups.

Group	Histological pathology of LEEP					The coincidence rate(%)	*X*^2^	*p*
	Chronic inflammation	LSIL	HSIL	Cervical cancer	Total			
ECC(−)&Biopsy(+)	109	126	314	8	557	57.81	142.367	<0.001
ECC(+)&Biopsy(−)	20	16	66	4	106	66.04		
ECC(+)&Biopsy(+)	21	24	386	52	483	90.68		

### 3.3. The consistency rate of ECC combined with colposcopic targeted biopsy and histological pathology of surgery was compared among the three groups

The consistency rate of ECC combined with colposcopic targeted biopsy and histological pathology of LEEP was compared among the three groups (Table 3). In “ECC(−)&Biopsy(+)” group, histological pathology of surgery was suggestive of HSIL+ in 57.81% of patients, of which 1.44% (8/557) indicated invasive carcinoma. In “ECC(+)&Biopsy(−)” group, 66.04% (70/106) of patients were confirmed to have HSIL+ after surgery, and the proportion of lesions that were cervical invasive carcinoma was 3.77% (4/106). In “ECC(+)&Biopsy(+)” group, histological pathology of surgery was suggestive of HSIL+ in 90.68% (438/483) of patients. Of these, 10.77% (52/483) were confirmed as having invasive carcinoma after surgery.

In the above three groups, the consistency rate of the results of surgical pathology and the detection rate of cervical invasive cancer in the “ECC(+)&Biopsy(+)” group were significantly higher than those in the other two groups (*p* < 0.05). However, there was no significant difference between the other two groups (*p* > 0.05).

### 3.4. LEEP resection margin and postoperative ECC positive rate in the three groups

The positive rate of LEEP resection margin and postoperative ECC were significantly different among the three groups (*p* < 0.05) (Table 4). In “ECC(−)&Biopsy(+)” group, the proportion of residual lesions after LEEP was 11.85% (66/557), significantly lower than the positive rate of ECC positive groups (41.43%) (p < 0.05). In “ECC(+)&Biopsy(+)” group, the proportion of residual lesions after LEEP was the highest, reaching 43.48% (210/483).

**Table 4:** The positive rate of surgical margin and ECC after LEEP in the three groups.

	Postoperative resection margin or postoperative ECC were positive		Positive rate(%)	*X*^2^	*p*
	Negative	Positive			
ECC negative	491	66	11.85	126.909	<0.001
ECC positive	345	244	41.43		

Group	Postoperative resection margin or postoperative ECC were positive		Positive rate(%)	*X*^2^	*p*
	Negative	Positive			
ECC(**−**)&BIOPSY(+)	491	66	11.85		
ECC(+)&BIOPSY(**−**)	72	34	32.08*	132.637	<0.001
ECC(+)&BIOPSY(+)	273	210	43.48*★		

*Compared with ECC negative group, the difference was statistically significant (*p* < 0.05).

★Compared with only the ECC positive group, the difference was statistically significant (*p* < 0.05).

## 4. Discussion

The progression from CIN to cervical cancer is a long process. Timely detection of precancerous cervical cancer lesions and active diagnosis and treatment can reduce the incidence of cervical cancer to a certain extent. When a patient is referred for colposcopy for abnormal cervical screening, ECC should be performed if there was a suspected lesion in the cervical canal (5, 9). However, the effectiveness and application indicators of ECC is still up for debate (7), and no clear consensus has been reached at present.

The positive rate of cervical lesions detected by ECC is inconsistent across the multiple studies undertaken. In one prospective study of 1,299 patients, there was a 72.4% diagnostic agreement between colposcopic targeted biopsy combined with ECC and specimens after cervical coning (10). In another study evaluating 150 patients with HSIL+ using the pathological results of postoperative conical specimens as the final criterion, colposcopic targeted biopsy combined with ECC had a 79% coincidence rate for a cervical HSIL+ diagnosis (11). In this current study, the diagnostic agreement rate of ECC combined with colposcopic targeted biopsy was higher than that of colposcopic targeted biopsy alone (72.43% vs. 67.54%). The extra detection rate of ECC for cervical HSIL+ was 6.11% (70/1146), which avoided the missed diagnosis of 70 patients with HSIL+, and four patients were confirmed to have cervical invasive carcinoma after LEEP surgery. As can be seen from our study data, when the patient has the following conditions, ECC can improve HSIL detection and reduce the missed diagnosis of HSIL when the patient is: high-grade cytology, HPV 16 infection, and colposcope impression suggestive of CIN3 + .

ECC combined with colposcopic targeted biopsy can help clinicians predict the extent of patients’ lesions and suggest follow-up treatment. The risk stratification of HSIL based on the results of targeted biopsy combined with ECC provides a reference for the choice of therapy for patients with HSIL who have fertility needs, which is of great significance for the protection of fertility.

In “ECC(−)&Biopsy(+)” group, the coincidence rate of ECC combined with colposcopic targeted biopsy and the pathological results after cervical coning was 57.81%. However, the rate of LEEP postoperatively confirmed that cervical invasive cancer was only 1.44%, and the positive rate of LEEP postoperative resection margin or ECC was the lowest in this group of patients, which was 11.85%. The above data suggests that if cervical HSIL was indicated only by targeted biopsy, not by ECC, 42.19% of patients were confirmed to have chronic inflammation or low-grade cervical lesions after surgery. This may be because the small HSIL lesions were removed at the time of biopsy. According to research, up to 20% of cervical HSIL patients have spontaneous regression after biopsy diagnosis, especially in young women and when the interval between biopsy and coning is longer (12, 13). These patients have a small possibility of being diagnosed with invasive cancer after surgery. For them, LEEP treatment was somewhat radical. Many studies have shown that cervical coning is associated with pregnancy-related conditions such as preterm birth, low birth weight, caesarean section and premature rupture of membranes, and intrauterine infections of the fetus (14, 15).

With reproductive health becoming the focus of social attention, protecting patients’ fertility has also become an important issue for clinicians. Therefore, for patients with fertility needs, can we adopt more conservative treatment when the ECC does not indicate HSIL? A study by Tan et al. (16) comparing the efficacy of focused ultrasound (FUS) and cryotherapy for cervical squamous intraepithelial lesions (SILs) showed that there was no significant difference in the efficacy of FUS and cryotherapy for patients with cervical HSILs (88.9% vs. 75.0%). In addition, at the follow-up of 6–12 months after treatment, there was no recurrence in HSIL patients treated with the above two methods. Noninvasive therapies such as topical Nocardia rubra cell wall skeleton and 5-ALA PDT are also increasingly used in the treatment of cervical HSIL and have shown good efficacy (17, 18). As much as 42.19% of the patients in “ECC(−)&Biopsy(+)” group had no HSIL lesions in the postoperative pathological specimens, which suggests that conservative treatment methods, such as Nocardia rubra cell wall skeleton and 5-ALA PDT, may be suitable for these patients. Furthermore, positive margins or ECC after LEEP were significantly associated with residual cervical canal lesions (19). The positive rate of LEEP resection margin and postoperative ECC in “ECC(−)&Biopsy(+)” group was low, which means that the conization depth can be reduced when we take the LEEP procedure, and there is no need to perform ECC for the residual cervical canal. This may be beneficial to patients as ECC can cause cervical canal adhesion, increased bleeding and adverse pregnancy outcomes (20). Therefore, our study provides a basis for this group of patients with cervical HSIL (HSIL on biopsy but no lesion on ECC) to choose conservative treatment and protect fertility.

When ECC indicated HSIL, the positive rate of LEEP resection margin and postoperative ECC was 41.43%, which was much higher than that in “ECC(−)&Biopsy(+)” group (41.43% vs. 11.85%) (*p* < 0.05). However, there were some differences between the “ECC(+)&Biopsy(−)” group and the “ECC(+)&Biopsy(+)” group.

When both ECC and targeted biopsy results suggested HSIL, the coincidence rate of targeted biopsy combined with ECC and the pathological results after cervical coning was 90.68%. Of these, 10.77% (52/483) were confirmed as having invasive carcinoma after surgery. The proportion of residual lesions after LEEP was 43.48%. Therefore, for these patients, we should adopt more active treatment (21), and the conization depth should be properly increased to prevent positive postoperative margins. ECC was performed immediately after conical resection to remind the possibility of residual lesions in the cervical canal. The postoperative management of patients should also be improved, and patients should be instructed to follow up regularly.

When only the ECC suggested HSIL, the consistency rate was 66.04%, while the LEEP-confirmed invasive cancer was only 3.77%, which was between the other two groups. For such patients, doctors can make treatment plans according to the patient’s age, HPV infection type, type of transformation area, and lesion size. It is worth noting that when the ECC result was higher than the pathological result of targeted biopsy, the positive rate of LEEP resection margin or ECC after surgery was 32.08%, which may be related to the type of lesion in the cervical canal. Therefore, during the operation, doctors should appropriately increase the depth of conization and perform ECC immediately after cervical coning to avoid a missed diagnosis in the residual cervical canal (21).

It is important to bear in mind the limitations of this study. First, this study was a retrospective, and one of the observational indicators was the positive rate of LEEP resection margin or ECC, so there may be artificial bias. Second, the 2019 ASCCP guidelines (22) already call on pathologists to distinguish HSIL as HSIL (CIN2) and HSIL (CIN3), because the bioethology of the two is inconsistent. For women with fertility requirements, CIN2 have more conservative options in management. However, the Department of Pathology in our hospital did not start to differentiate CIN2 and CIN3 for HSIL until 2021, which means we were unable to differentiate the patients’ pathological findings in this study. In addition, the sample size of ECC higher than the multipoint biopsy pathology group was small. More prospective studies with high quality and larger sample sizes should be designed in the future.

In summary, this study shows that ECC combined with colposcopic targeted biopsy is necessary. ECC can improve the detection rate of cervical HSIL and reduce missed diagnosis. And clinicians are able to risk stratify HSIL patients according to ECC, so as to make a decision for subsequent treatment. The result showed great potential for the protection of patient fertility.

## Data availability statement

The raw data supporting the conclusions of this article will be made available by the authors, without undue reservation.

## Ethics statement

The studies involving human participants were reviewed and approved by the Ethics Committee of The First Affiliated Hospital of Chongqing Medical University. The Ethics Committee waived the requirement of written informed consent for participation.

## Author contributions

JY and LS contributed to the conception of the study. LS, WJ, LM, and DZ performed the experiment. LS and LX contributed significantly to analysis and manuscript preparation. LS performed the data analyses and wrote the manuscript. JY, WJ, LM, and DZ helped perform the analysis with constructive discussions. All authors contributed to the article and approved the submitted version.

## Conflict of interest

The authors declare that the research was conducted in the absence of any commercial or financial relationships that could be construed as a potential conflict of interest.

## Publisher’s note

All claims expressed in this article are solely those of the authors and do not necessarily represent those of their affiliated organizations, or those of the publisher, the editors and the reviewers. Any product that may be evaluated in this article, or claim that may be made by its manufacturer, is not guaranteed or endorsed by the publisher.

## References

[1] Wang B Su Y Zhang C Zhou M Yuan S Zhang M. The effect of local photodynamic therapy with 5-aminolevulinic acid in treating different grades of cervical intraepithelial neoplasia. Photodiagn Photodyn Ther. (2022) 40:103196. doi: 10.1016/j.pdpdt.2022.103196 36368451

[2] Singh D Vignat J Lorenzoni V Eslahi M Ginsburg O Lauby-Secretan B. Global estimates of incidence and mortality of cervical cancer in 2020: a baseline analysis of the who global cervical cancer elimination initiative. Lancet Glob Health. (2022) 11:e197–206. doi: 10.1016/S2214-109X(22)00501-0, PMID: 36528031 PMC9848409

[3] Kyrgiou M Arbyn M Bergeron C Bosch FX Dillner J Jit M. Cervical screening: esgo-efc position paper of the european society of gynaecologic oncology (esgo) and the european federation of colposcopy (efc). Br J Cancer. (2020) 123:510–7. doi: 10.1038/s41416-020-0920-9, PMID: 32507855 PMC7434873

[4] Ren H Jia M Zhao S Li H Fan S. Factors correlated with the accuracy of colposcopy-directed biopsy: a systematic review and meta-analysis. J Invest Surg. (2022) 35:284–2. doi: 10.1080/08941939.2020.1850944, PMID: 33377808

[5] Massad LS Perkins RB Naresh A Nelson EL Spiryda L Gecsi KS. Colposcopy standards: guidelines for endocervical curettage at colposcopy. J Low Genit Tract Dis. (2023) 27:97–1. doi: 10.1097/LGT.0000000000000710, PMID: 36222824 PMC9770112

[6] Klam S Arseneau J Mansour N Franco E Ferenczy A. Comparison of endocervical curettage and endocervical brushing. Obstet Gynecol. (2000) 96:90–4. doi: 10.1016/s0029-7844(00)00836-x, PMID: 10862849

[7] Driggers RW Zahn CM. To ecc or not to ecc: the question remains. Obstet Gynecol Clin N Am. (2008) 35:583–7. doi: 10.1016/j.ogc.2008.09.007 19061818

[8] Wu Q Jiang Y Ding J Xia L Xu H. Clinical predictors of residual disease in hysterectomy following a loop electrosurgical excision procedure for cervical intraepithelial neoplasia grade 3. BMC Pregnancy Childbirth. (2022) 22:1–6. doi: 10.1186/s12884-022-05281-y, PMID: 36575366 PMC9793502

[9] Wright TC Massad LS Dunton CJ Spitzer M Wilkinson EJ Solomon D. 2006 consensus guidelines for the management of women with abnormal cervical screening tests. J Low Genit Tract Dis. (2007) 11:201–2. doi: 10.1097/LGT.0b013e3181585870, PMID: 17917566

[10] Li Y Gong Y-X Wang Q Gao S Zhang H Xie F. Optimizing the detection of occult cervical cancer: a prospective multicentre study in China. Int J Women's Health. (2021) 13:1005–15. doi: 10.2147/IJWH.S329129, PMID: 34737649 PMC8558636

[11] Rubeša-Mihaljević R Vrdoljak-Mozetič D Dinter M Verša Ostojić D Štemberger-Papić S Klarić M. Diagnostic three slides pap test compared to punch biopsy and endocervical curettage in confirmed hsil+ diagnosis. Diagn (Basel, Switzerland). (2021) 11:942. doi: 10.3390/diagnostics11060942, PMID: 34070458 PMC8229939

[12] Trimble CL Piantadosi S Gravitt P Ronnett B Pizer E Elko A. Spontaneous regression of high-grade cervical dysplasia: effects of human papillomavirus type and hla phenotype[J]. Clin Cancer Res. (2005) 11:4717–23. doi: 10.1158/1078-0432.CCR-04-2599, PMID: 16000566 PMC3132609

[13] Guo Y Wang Y Peng Q Li L Zou M Wang C. Absence of high-grade cervical intraepithelial neoplasia in conization specimens from patients with colposcopic biopsy-confirmed high-grade cervical intraepithelial neoplasia: retrospective study of 1695 cases. Front Oncol. (2022) 12:980884. doi: 10.3389/fonc.2022.980884, PMID: 36185239 PMC9515539

[14] Kyrgiou M Athanasiou A Paraskevaidi M Mitra A Kalliala I Martin-Hirsch P. Adverse obstetric outcomes after local treatment for cervical preinvasive and early invasive disease according to cone depth: systematic review and meta-analysis. BMJ (Clinical Research Ed). (2016) 354:i3633. doi: 10.1136/bmj.i3633, PMID: 27469988 PMC4964801

[15] Kacerovsky M Musilova I Baresova S Kolarova K Matulova J Wiik J. Cervical excisional treatment increases the risk of intra-amniotic infection in subsequent pregnancy complicated by preterm prelabor rupture of membranes. Am J Obstet Gynecol. (2022) 229:51.e1–51.e13. doi: 10.1016/j.ajog.2022.12.316 36596440

[16] Tan R Xiao L Sun J Liu M Zhang X Chang S. A retrospective study of focused ultrasound versus cryotherapy in treatment of cervical squamous intraepithelial lesions. Int J Hypertherm. (2022) 39:1294–9. doi: 10.1080/02656736.2022.2129104, PMID: 36191925

[17] Zhao J Feng H Wang T Pang X Zhou Y Cui Y. The safety and efficacy of a novel method for treatment of hsil. Arch Gynecol Obstet. (2021) 304:1291–8. doi: 10.1007/s00404-021-06047-1 33813597

[18] Qu Z Wang Z Qiu S Cui G Li C. Efficacy of photodynamic therapy with 5-aminolevulinic acid for the treatment of cervical high-grade squamous intraepithelial lesions with high-risk hpv infection: a retrospective study[J]. Photodiagn Photodyn Ther. (2022) 40:103068. doi: 10.1016/j.pdpdt.2022.103068 36002107

[19] Lang L Jia Y Duan Z Wu J Luo M Tian P. The role of endocervical curettage in detection and treatment of cervical canal lesions[J]. Histol Histopathol. (2022) 37:63–8. doi: 10.14670/HH-18-394, PMID: 34755328

[20] Massad LS. Selecting patients for endocervical curettage. J Low Genit Tract Dis. (2015) 19:271–2. doi: 10.1097/LGT.0000000000000130, PMID: 26125095

[21] An J Lei H Xie X Sun P. An abnormal precone endocervical curettage result is an independent risk factor for positive margins in conization specimens[J]. Oncol Res Treat. (2020) 43:518–5. doi: 10.1159/000509254, PMID: 32772026

[22] Perkins RB Guido RS Castle PE Chelmow D Einstein MH Garcia F. 2019 asccp risk-based management consensus guidelines for abnormal cervical cancer screening tests and cancer precursors. J Low Genit Tract Dis. (2020) 24:102–1. doi: 10.1097/LGT.0000000000000525, PMID: 34542089

